# Supplementary material for: Asynchronous magnetic resonance elastography: Shear wave speed reconstruction using noise correlation of incoherent waves
Source: Magn Reson Med. 2022 Oct 27;89(3):990–1001. doi: 10.1002/mrm.29502 (PMC9792433; doi:10.1002/mrm.29502)
Supplement: Supplementary file 1 — DATA S1 MATLAB code used to generate the numerical simulations of Figure 1 [file MRM-89-990-s002.zip › k-Wave/helpfiles/k-wave_elastic_wave_propagation.html]

Elastic Wave Propagation (k-Wave)


# Elastic Wave Propagation

- Example: Explosive Source In A Layered Medium
- Example: Plane Wave Absorption
- Example: Shear Waves And Critical Angle Reflection
- Example: Simulations In Three Dimensions
